# Supplementary material for: An FDA-Approved Antifungal, Ketoconazole, and Its Novel Derivative Suppress tGLI1-Mediated Breast Cancer Brain Metastasis by Inhibiting the DNA-Binding Activity of Brain Metastasis-Promoting Transcription Factor tGLI1
Source: Cancers (Basel). 2022 Aug 31;14(17):4256. doi: 10.3390/cancers14174256 (PMC9454738; doi:10.3390/cancers14174256)

Figure 1

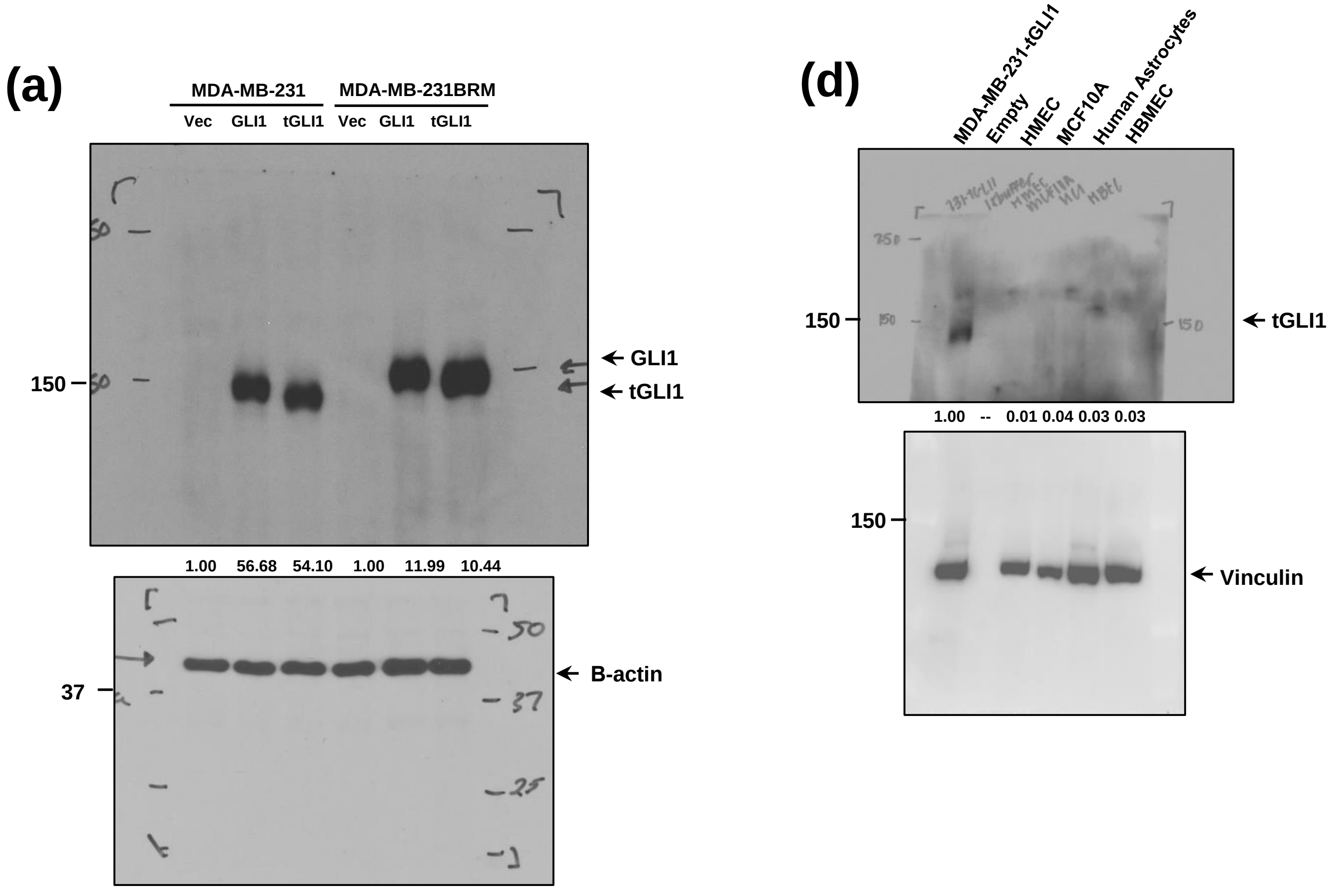

Figure 2

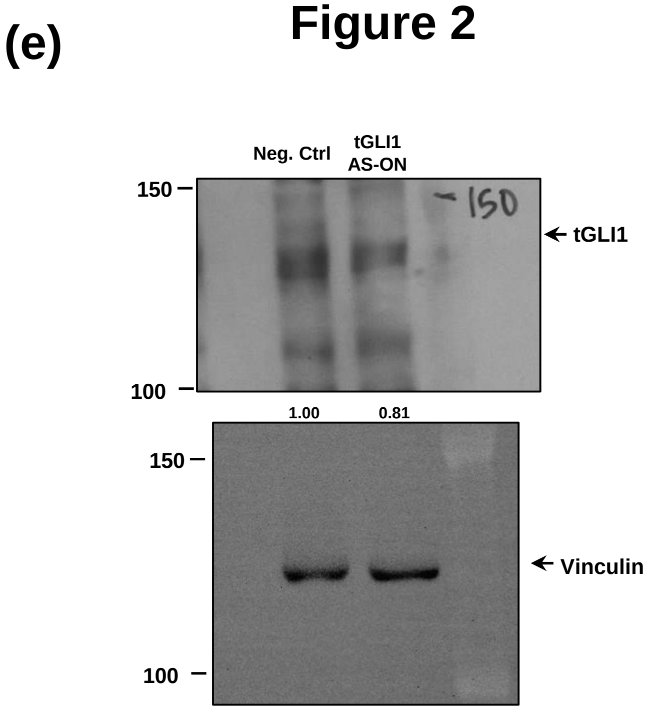

Figure 6

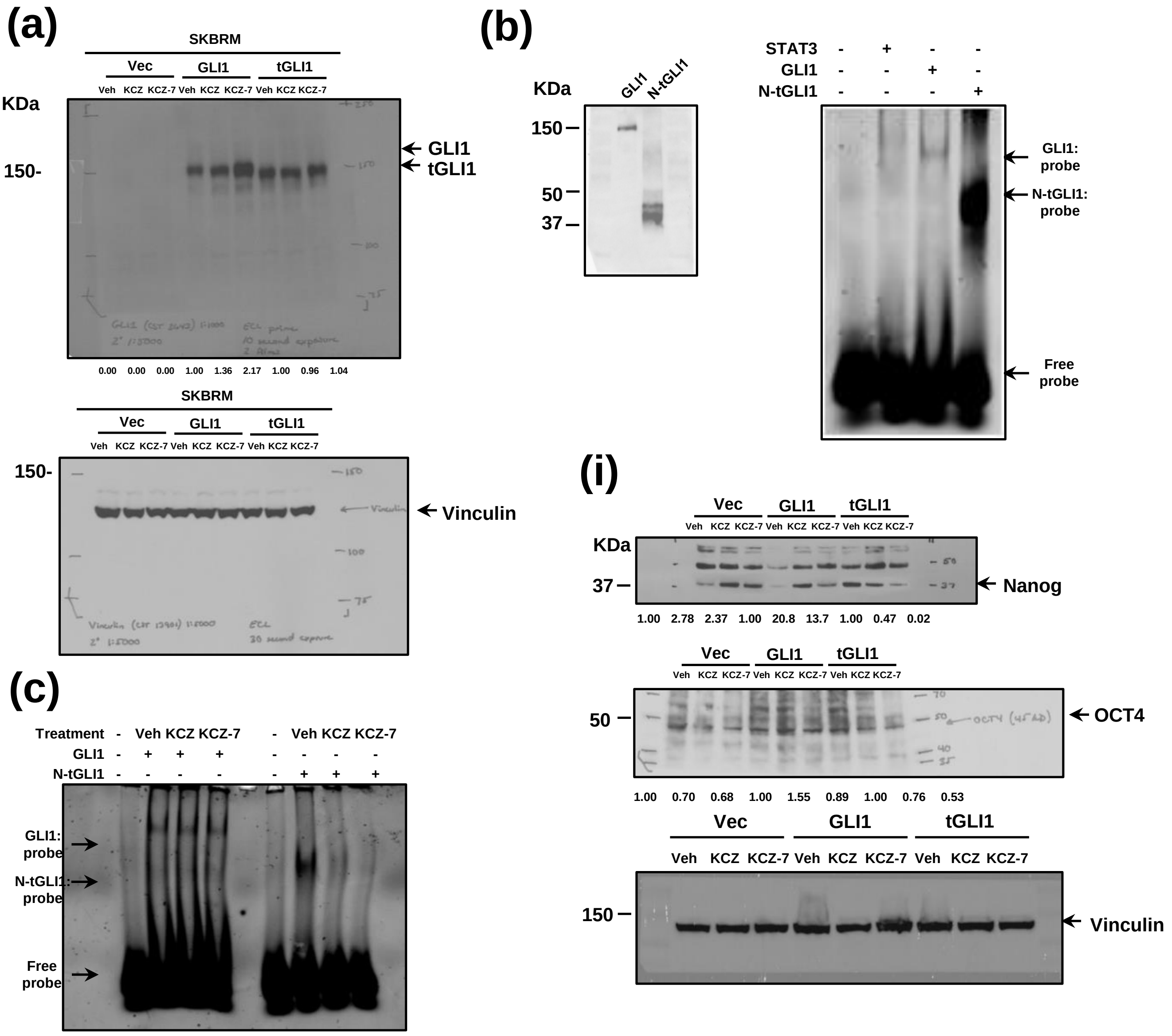

(a)
SKBRM
Vec
GLI1
tGLI1
Veh KCZ KCZ-7 Veh KCZ KCZ-7 Veh KCZ KCZ-7
KDa
150-
GLI1
tGLI1
0.00 0.00 0.00 1.00 1.36 2.17 1.00 0.96 1.04
SKBRM
Vec
GLI1
tGLI1
Veh KCZ KCZ-7 Veh KCZ KCZ-7 Veh KCZ KCZ-7
150-
Vinculin
(b)
KDa
GLI1
N-tGLI1
150
50
37
STAT3 - + - -
GLI1 - - + -
N-tGLI1 - - - +
GLI1: probe
N-tGLI1: probe
Free probe
(c)
Treatment - Veh KCZ KCZ-7 - Veh KCZ KCZ-7
GLI1 - + + + - - - -
N-tGLI1 - - - - - + + +
GLI1: probe
N-tGLI1: probe
Free probe
(i)
Vec
GLI1
tGLI1
Veh KCZ KCZ-7 Veh KCZ KCZ-7 Veh KCZ KCZ-7
KDa
37
Nanog
1.00 2.78 2.37 1.00 20.8 13.7 1.00 0.47 0.02
Vec
GLI1
tGLI1
Veh KCZ KCZ-7 Veh KCZ KCZ-7 Veh KCZ KCZ-7
50
OCT4
1.00 0.70 0.68 1.00 1.55 0.89 1.00 0.76 0.53
Vec
GLI1
tGLI1
Veh KCZ KCZ-7 Veh KCZ KCZ-7 Veh KCZ KCZ-7
150
Vinculin

Supplementary Figure S8

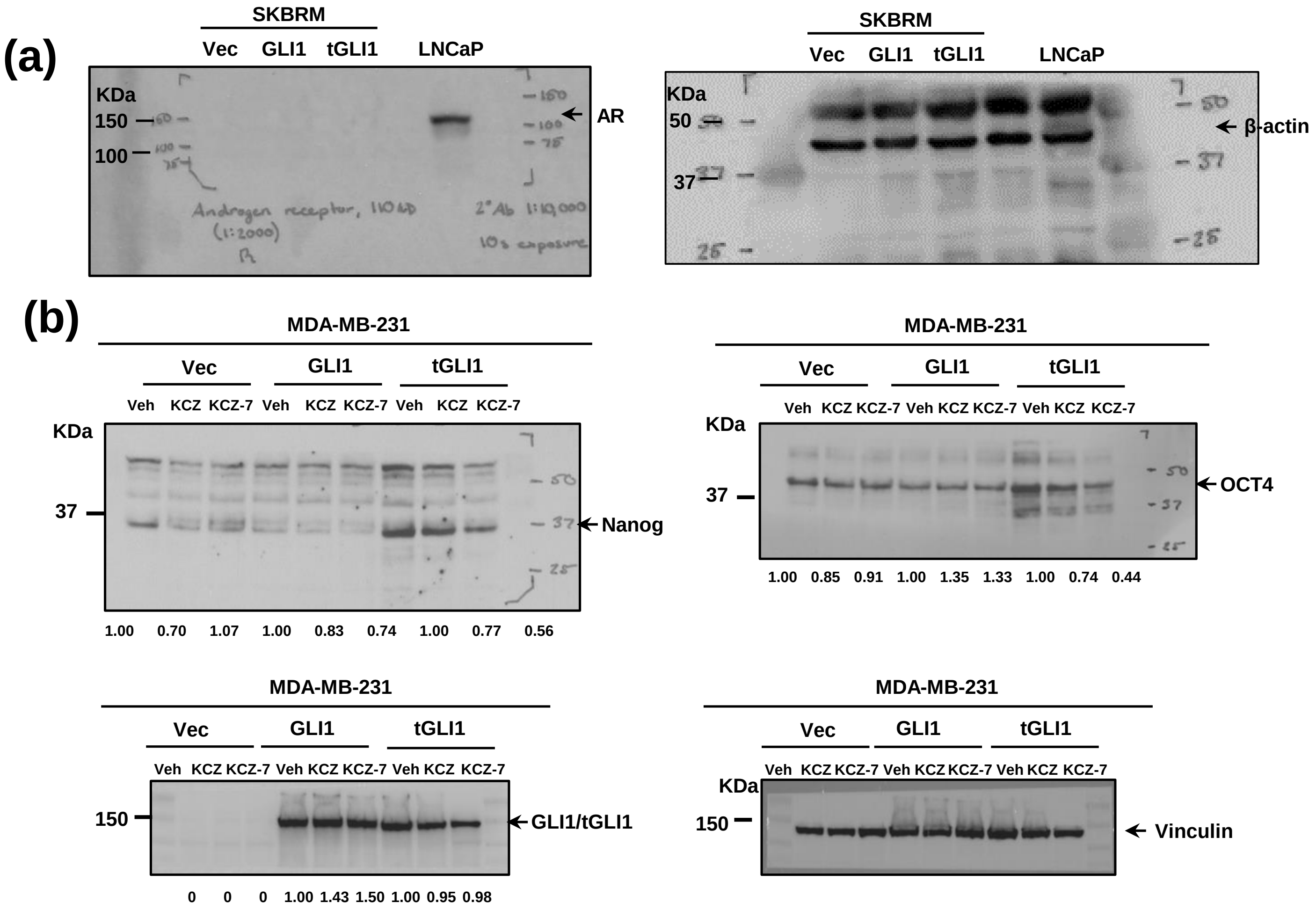

Supplementary Figure S12

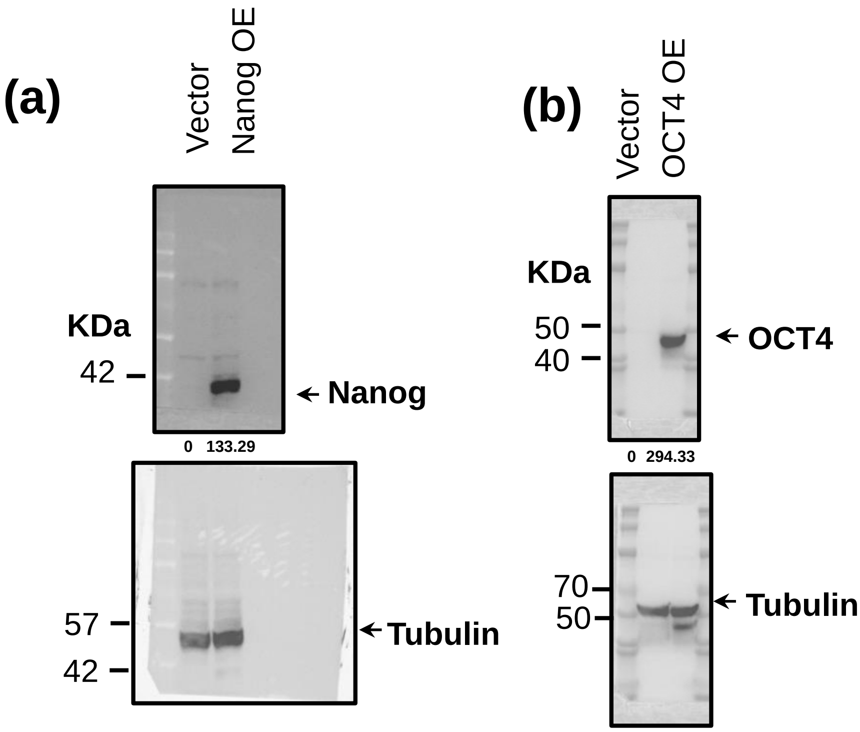

Supplementary Figure S13

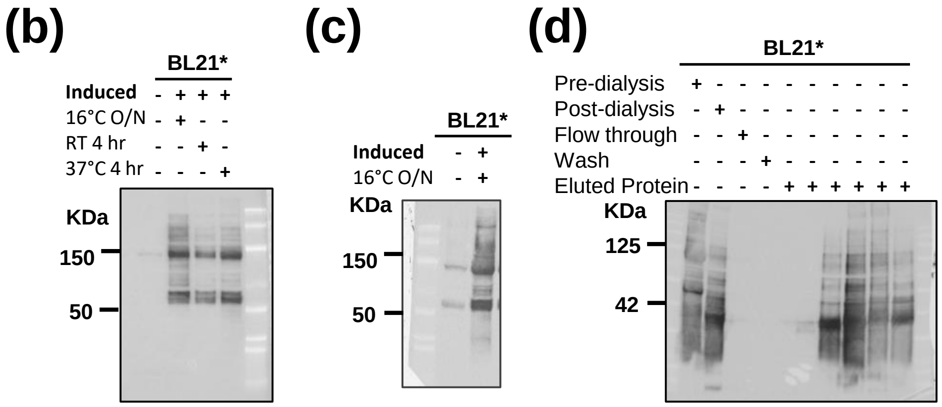

Supplement: Supplementary file 1 [file cancers-14-04256-s001.zip › File S1-Western blot uncropped images.pdf]
