# Supplementary material for: An FDA-Approved Antifungal, Ketoconazole, and Its Novel Derivative Suppress tGLI1-Mediated Breast Cancer Brain Metastasis by Inhibiting the DNA-Binding Activity of Brain Metastasis-Promoting Transcription Factor tGLI1
Source: Cancers (Basel). 2022 Aug 31;14(17):4256. doi: 10.3390/cancers14174256 (PMC9454738; doi:10.3390/cancers14174256)
Supplement: Supplementary file 1 [file cancers-14-04256-s001.zip › Suppl. Figures and Table S1.pdf]

Supplementary Figure S1

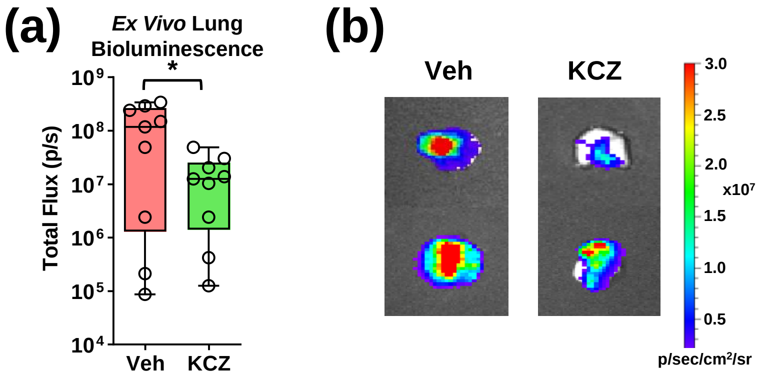

Supplementary Figure S1. KCZ treatment reduces lung engraftment of tGLI1-positive breast cancer cells. Related to Figure 3.
(**a**) *Ex vivo* lung bioluminescence at study endpoint. (**b**) Representative *ex vivo* lung bioluminescence images at study endpoint. *, $P < 0.05$; two-tailed Student's *t*-test was used to calculate p-value.

Supplementary Figure S2

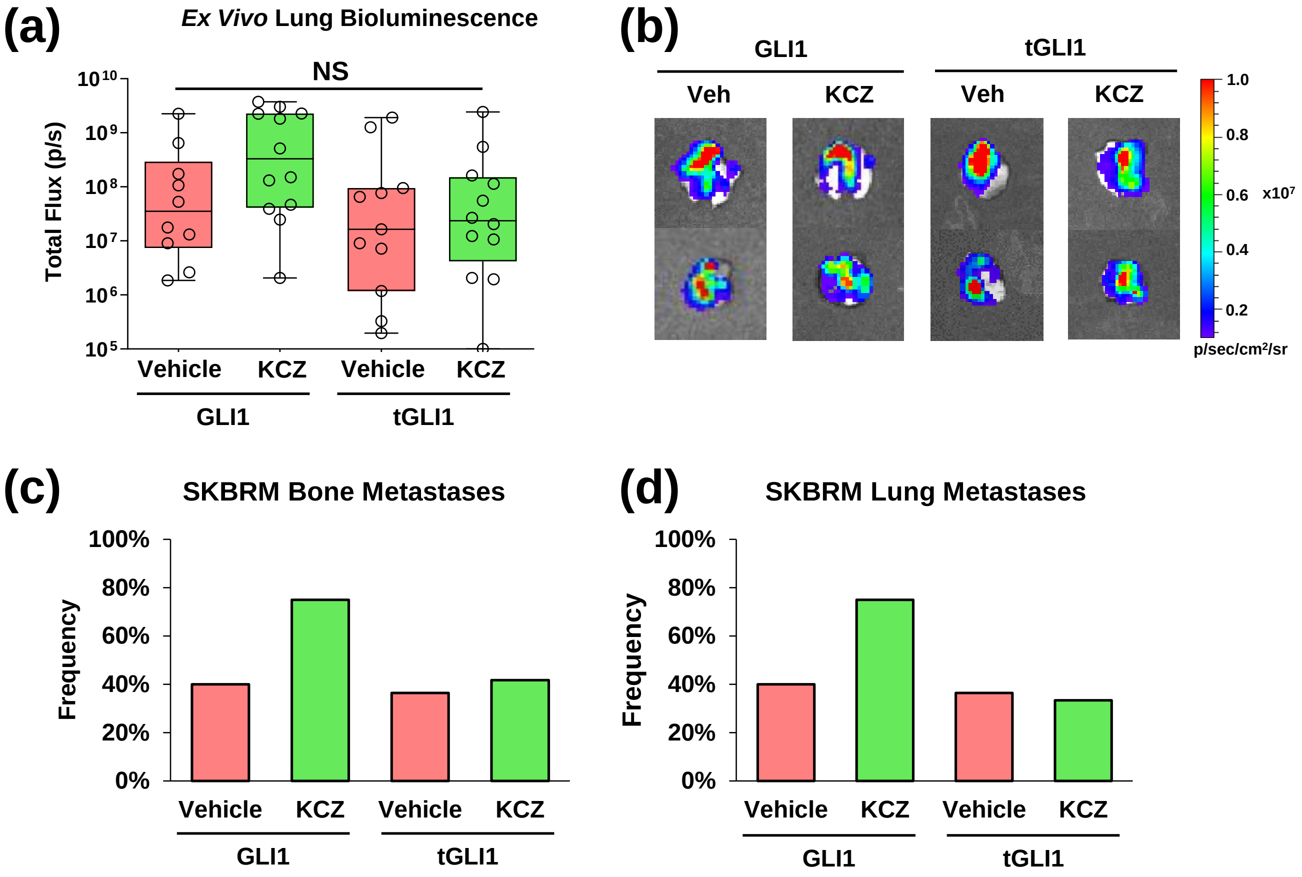

Supplementary Figure S2. KCZ treatment does not reduce progression of tGLI1-positive breast cancer lung metastases *in vivo*. Related to Figure 4.
(**a**) *Ex vivo* lung bioluminescence at study endpoint. (**b**) Representative *ex vivo* lung bioluminescence images. (**c**) Bone metastasis incidence. (**d**) Lung metastasis incidence. NS, not significant; two-way ANOVA with *post hoc* Bonferroni's multiple comparison test was used to calculate p-value.

Supplementary Figure S3

(a)

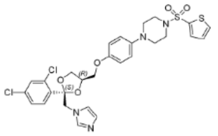

KCZ-3

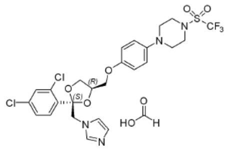

KCZ-4

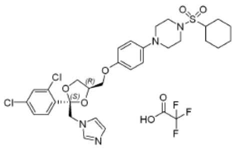

KCZ-5

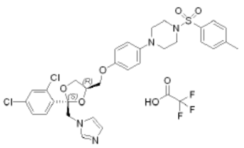

KCZ-6

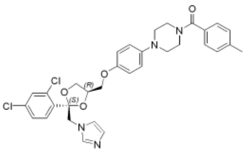

KCZ-7

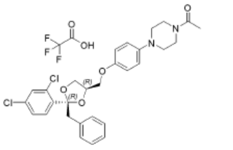

KCZ-10

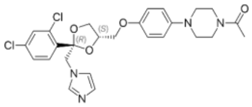

KCZ

(b)

KCZ-3

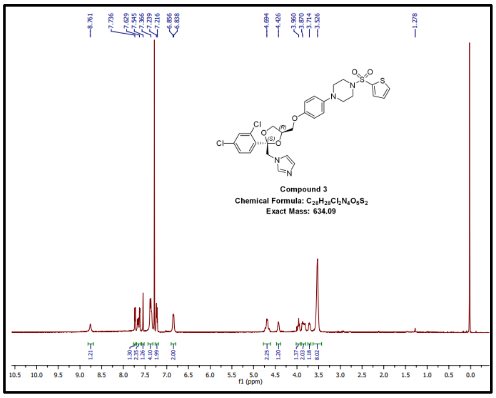

KCZ-4

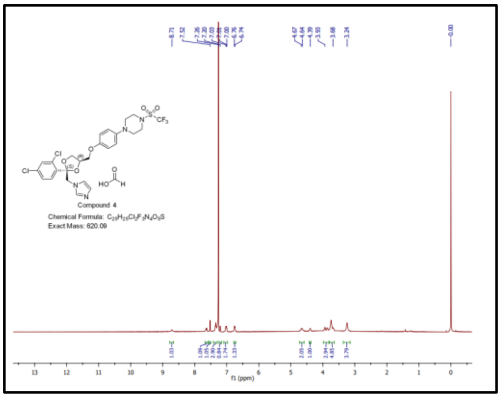

KCZ-5

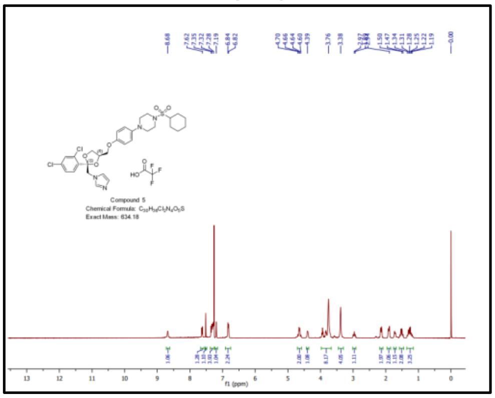

KCZ-6

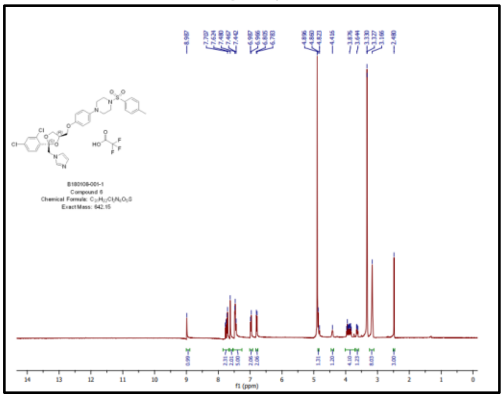

KCZ-7

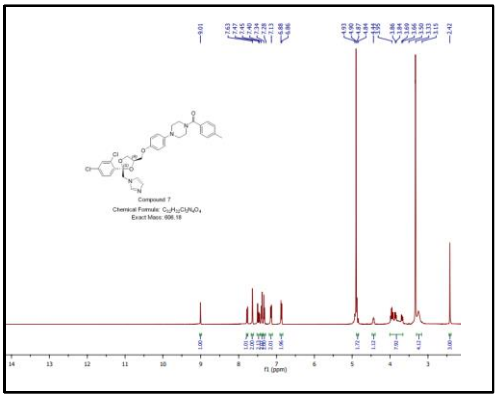

KCZ-10

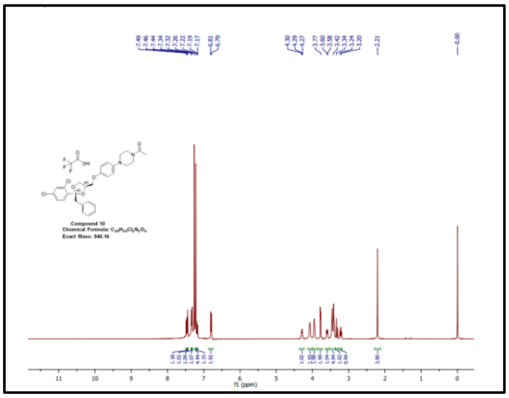

Supplementary Figure S3. Chemical structures of novel KCZ derivatives. Related to Figure 5. (**a**) Structures of novel KCZ derivatives and KCZ. (**b**) ^{1}H-NMR spectra for novel KCZ derivatives.

Supplementary Figure S4

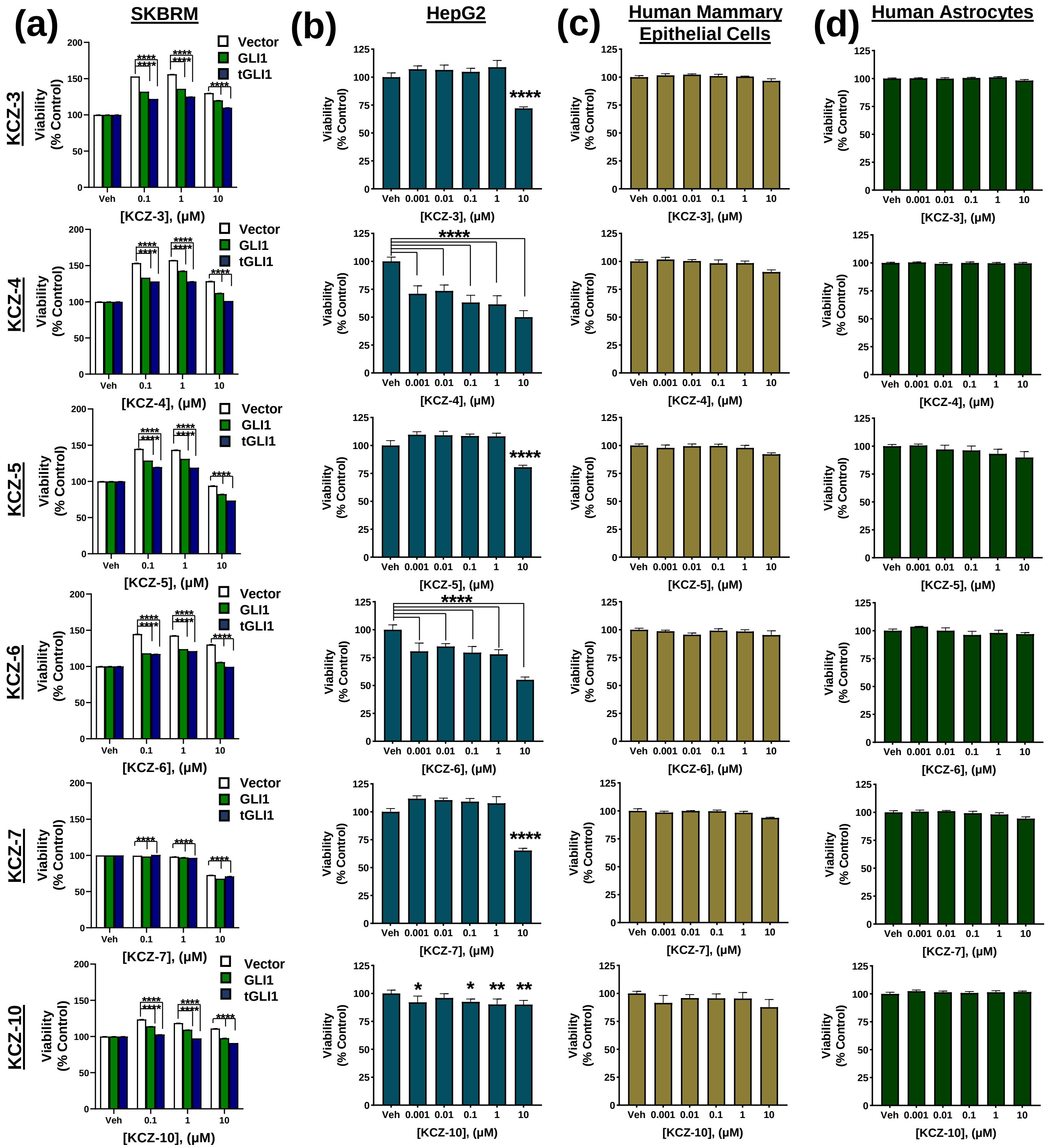

Supplementary Figure S4. Novel KCZ derivatives elicit little to no toxicity in HepG2, human mammary epithelial cells, and human astrocytes. Related to Figure 5.
(**a**) Cell viability assay using SKBRM cells stably expressing either control vector, GLI1-, or tGLI1-expression vectors. (**b-d**) Viability assay using hepatocellular carcinoma (HepG2) cells (**b**)**,** human mammary epithelial cells (**c**), and human astrocytes. (**a**) *, $P < 0.05$; **, $P < 0.01$; ****, $P < 0.0001$; two-way ANOVA with *post hoc* Dunnett's multiple comparison test and (**d**) one-way ANOVA with *post hoc* Bonferroni's multiple comparison test (**b-d**) was used to calculate p-values.

Supplementary Figure S5

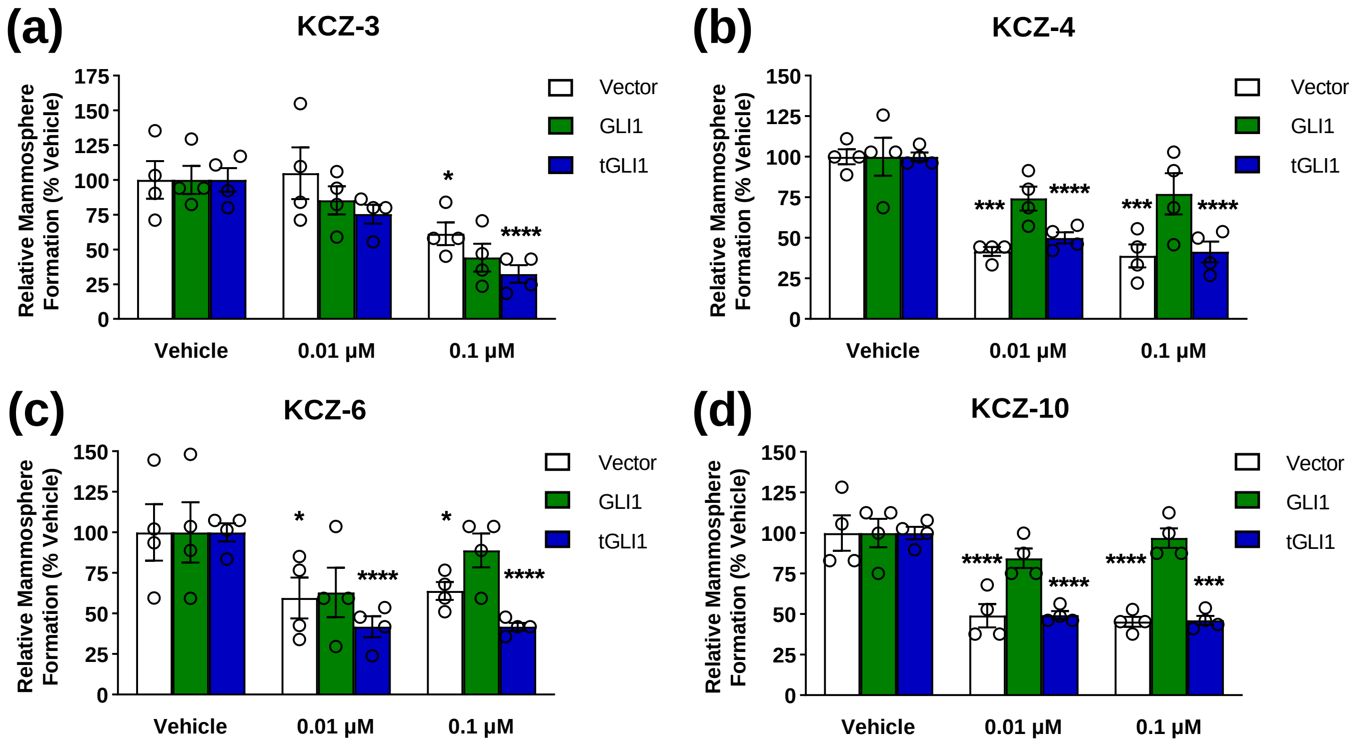

Supplementary Figure S5. Novel KCZ derivatives. Related to Figure 5.
(**a-d**) Non-selective inhibition of SKBRM mammosphere formation. Comparisons relative to the vehicle control for each cell line. *, *P* < 0.05; ***, *P* < 0.001; ****, *P* < 0.0001; two-way ANOVA with *post hoc* Dunnett's multiple comparison test (**a-d**) was used to calculate p-values.

Supplementary Figure S6

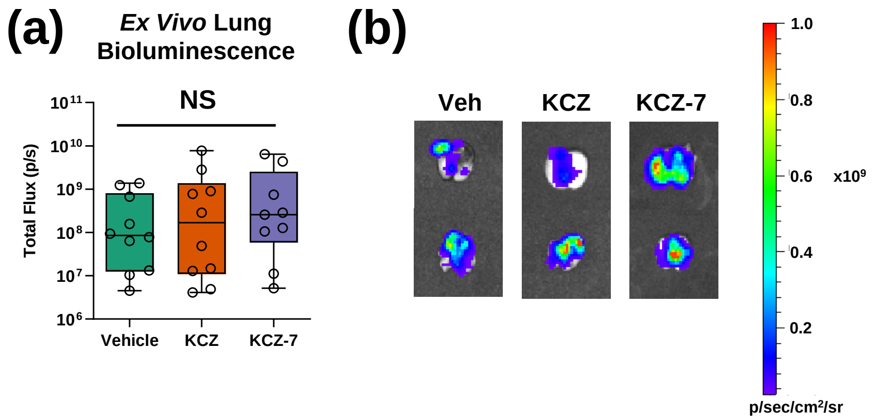

Supplementary Figure S6. KCZ-7 does not reduce SKBRM-tGLI1 lung metastasis progression. Related to Figure 5.

(**a**) *Ex vivo* lung bioluminescence at study endpoint (N = 10 per group). (**b**) Representative *ex vivo* lung bioluminescence images at study endpoint. NS, not significant; one-way ANOVA with *post hoc* Tukey's multiple comparison test was used to calculate p-values.

Supplementary Figure S7

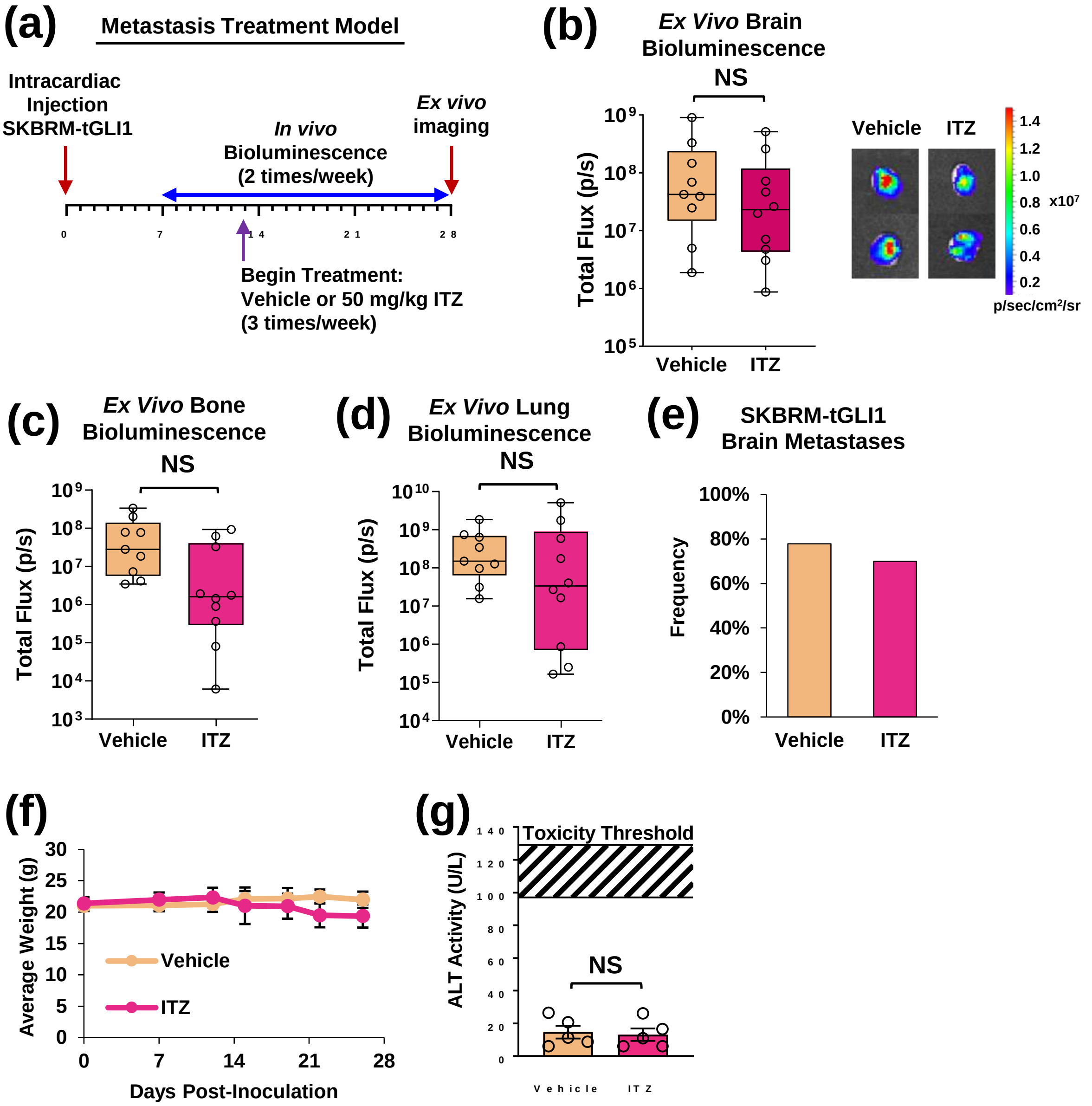

Supplementary Figure S7. ITZ does not reduce progression of SKBRM-tGLI1 metastases *in vivo*. Related to Figure 5.

(**a**) Schema for intracardiac brain metastasis treatment model. (**b-d**) *Ex vivo* bioluminescence of resected brain (**b**), bone (**c**) and lungs (**d**) at study endpoint (*N* = 9-10 per group). (**e**) Incidence of brain metastases at study endpoint. (**f**) Average weight of mice treated with vehicle or 50 mg/kg Itraconazole (ITZ). (**g**) Serum alanine transaminase (ALT) activity. Striped region represents range of ALT activity in athymic mice following thioacetamide-induced acute liver injury. NS, not significant; two-tailed Student's *t*-test was used to calculated p-values.

Supplementary Figure S8

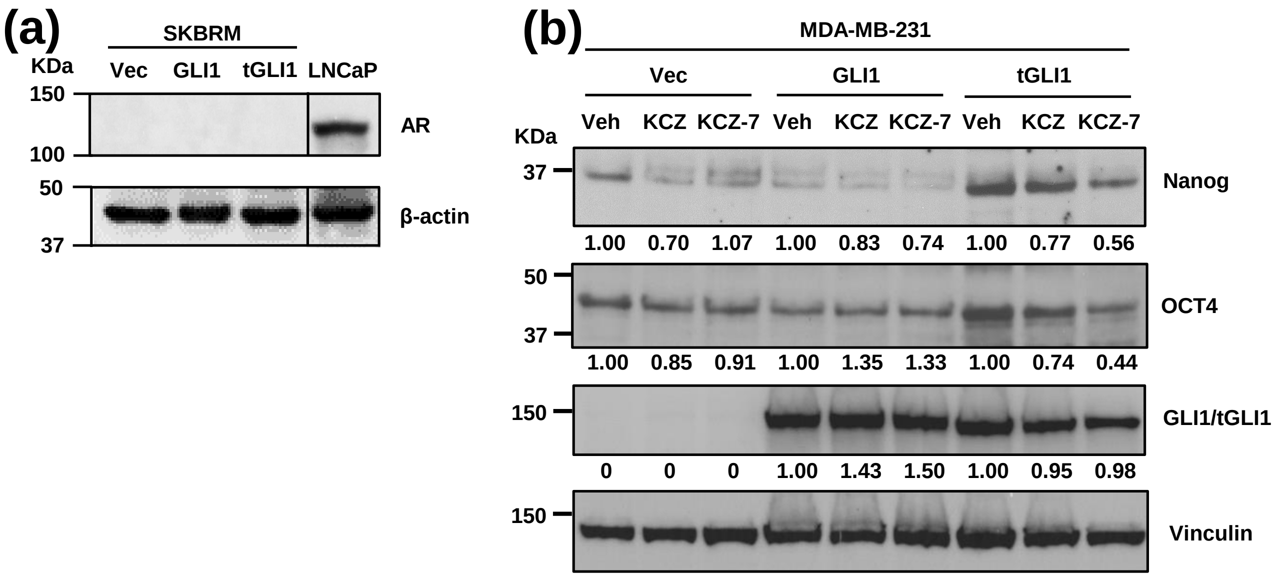

Supplementary Figure S8. KCZ and KCZ-7 treatment reduce expression of tGLI1-mediated stemness genes *Nanog* and *OCT4* in MDA-MB-231 cells. Related to Figure 6.
(**a**) Androgen receptor (AR) expression in isogenic SKBRM cell lines. LNCaP, a metastatic prostate cancer cell line, was used as a positive control. (**b**) Representative western blot of Nanog, OCT4, GLI1, and tGLI1 expression in isogenic MDA-MB-231 cell lines following 24 h treatment with vehicle, 1 μM KCZ, or 1 μM KCZ-7. The same membrane was probed to assess the loading control.

Supplementary Figure S9

```
1          11         21         31         41         51
MFNSMTPPPI SSYGEPCCLR PLPSQGAPSV GTEVKLTKKR ALSISPLSDA SLDLQTVIRT

SPSSLVAFIN SRCTSPGGSY GHLSIGTMSP SLGFPAQMNH QKGPSPSFGV QPCGPHDSAR

GGMIPHPQSR GPFPTCQLKS ELDMLVGKCR EEPLEGDMSS PNSTGIQDPL LGMLDGREDL

EREEKREPES VYETDCRWDG CSQEFDSQEQ LVHHINSEHI HGERKEFVCH WGGCSRELRP

FKAQYMLVVH MRRHTGEKPH KCTFEGCRKS YSRLENLKTH LRSHTGEKPY MCEHEGCSKA

FSNASDRAKH QNRTHSNEKP YVCKLPGCTK RYTDPSSLRK HVKTVHGPDA HVTKRHRGDG

PLPRAPSIST VEPKREREGG PIREESRLTV
```

Molecular weight: 40.87 kDa
Length: 352/1065 AA

Supplementary Figure S9. Sequence of recombinant N-tGLI1 protein as determined by mass spectrometry. Related to Figure 6.
The first 390 amino acids of the tGLI1 protein sequence are shown. Residues that form the zinc finger domains are in bold. Highlighted residues were detected by mass spectrometry.

Supplementary Figure S10

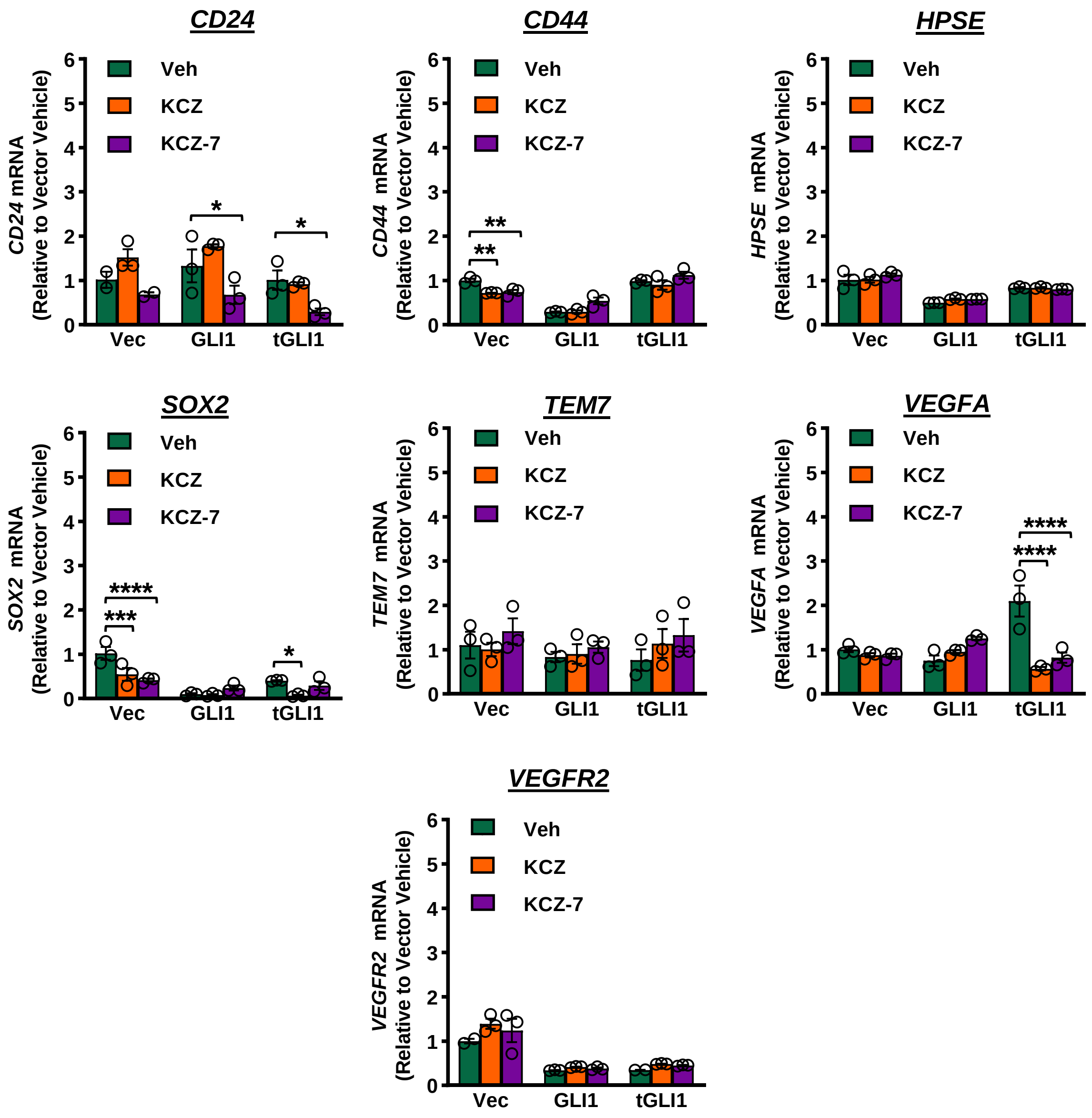

Supplementary Figure S10. Expression of validated tGLI1 target genes after treatment with KCZ or KCZ-7 in isogenic SKBRM cell lines. Related to Figure 6.
Expression of validated tGLI1 target genes in isogenic SKBRM lines following 24 h treatment with vehicle, 1 µM KCZ, or 1 µM KCZ-7 as assessed by RT-qPCR. *, $P < 0.05$; ** $P < 0.01$; ***, $P < 0.001$; ****, $P < 0.0001$; two-way ANOVA with Bonferroni's multiple comparison test was used to calculate p-values.

Supplementary Figure S11

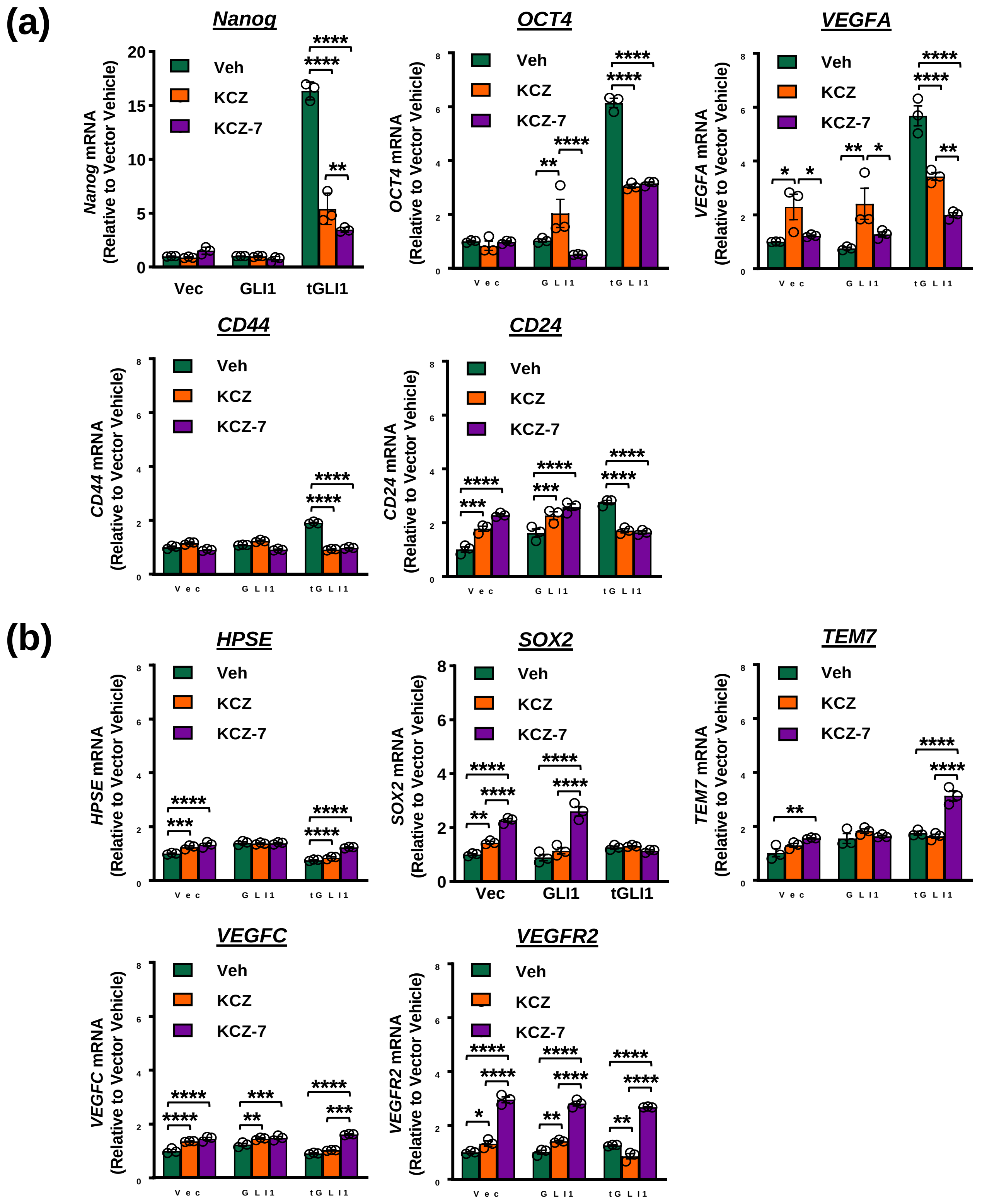

Supplementary Figure S11. Expression of validated tGLI1 target genes after treatment with KCZ or KCZ-7 in isogenic MDA-MB-231 cell lines. Related to Figure 6.
(**a-b**) Expression of validated tGLI1 target genes in MDA-MB-231 stable lines following 24 h treatment with vehicle, 1 μM KCZ, or 1 μM KCZ-7 as assessed by RT-qPCR. *, $P < 0.05$; ** $P < 0.01$; ***, $P < 0.001$; ****, $P < 0.0001$; two-way ANOVA with Bonferroni's multiple comparison test was used to calculate p-values.

Supplementary Figure S12

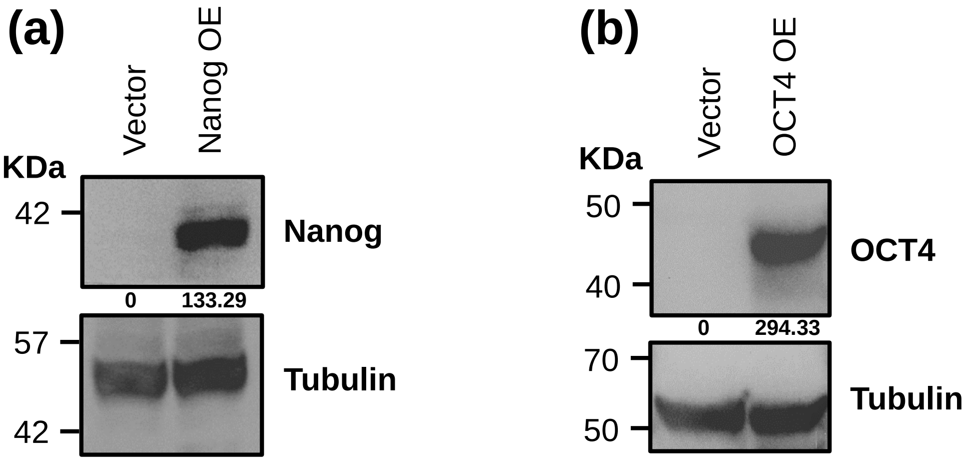

Supplementary Figure S12. Nanog and OCT4 overexpression in transfected SKBRM-tGLI1 cells. Related to Figure 6.
(**a-b**) Overexpression of Nanog (**a**) and OCT4 (**b**) in transfected SKBRM-tGLI1 cells. OE, overexpression.

Supplementary Figure S13

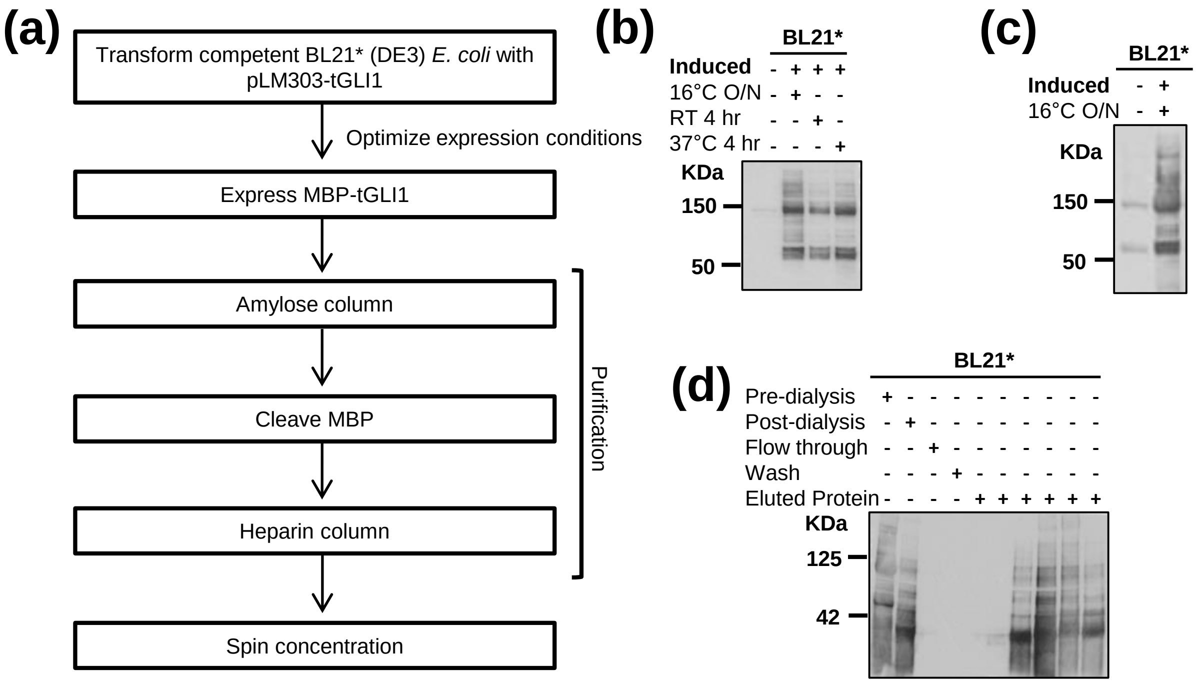

Supplementary Figure S13. pLM303-tGLI1 recombinant protein purification.
(**a**) Schematic of pLM303-tGLI1 recombinant protein purification. (**b**) tGLI1 protein expression after competent *E. coli* strain, BL21* (DE3), was transformed and induced with 0.3 mM IPTG at 16°C overnight (O/N), room temperature (RT), or 37°C for expression optimization. BL21* (DE3) *E. coli* was transformed, expanded, induced with 0.3 mM IPTG at 16°C, homogenized for lysis, and clear lysate was passed over amylose resin high flow (**c**). The desired fractions were collected and dialyzed O/N containing HRV 3C PreScission Protease to cleave the MBP tag. Purified tGLI1 recombinant protein was separated from cleaved MBP using Heparin HiTrap (**d**). Eluted tGLI1 recombinant protein was spin concentrated and frozen on dry ice for further use.

Supplementary Table S1

Supplementary Table S1. RT-qPCR primers.

Gene	Forward sequence (5'-3')	Reverse Sequence (5'-3')
CD24	CTGCTCCTACCCACGCACATT	GGCCAACCCAGAGTTGGAAGT
CD44	TCAGAGGAGTAGGAGAGAGGAAAC	AAGTCAAAGTAACAATAAGAGTGGTCA
GLI1	CACCAAGCTAACCTCATGTC	CGGGGAGAAGAAAAGAGTGGG
HPSE	TACCTTCATTGCACAAACACTG	ACTTGGTGACATTATGGAGGTT
Nanog	CTAAGAGGTGGCAGAAAAACA	CTGGTGGTAGGAAGAGTAAAGG
OCT4	TGGTCCGAGTGTGGTTCTGTAA	TGTGCATAGTCGCTGCTTGAT
SOX2	GGAGTTGTCAAGGCAGAGAAGAG	GAGAGAGGCAAACTGGAATC
tGLI1	GTGTGGGGACAGAAGTCAA	GTGCGGATAACCGTCTGC
TEM7	CTTCAACCCTGGCTACTCCG	GCTGATTTCCGGGACAGACA
VEGFA	TGTCTAATGCCCTGGAGCCT	TTAACTCAAGCTGCCTCGCC
VEGFR2	GCATCCAGTGGGCTGATGAC	GCAGGGATTCTGACACACTC
GAPDH	ACTGCCAACGTGTCAGTGG	GTGTCGCTGTTGAAGTCAGA
