# Supplementary material for: An FDA-Approved Antifungal, Ketoconazole, and Its Novel Derivative Suppress tGLI1-Mediated Breast Cancer Brain Metastasis by Inhibiting the DNA-Binding Activity of Brain Metastasis-Promoting Transcription Factor tGLI1
Source: Cancers (Basel). 2022 Aug 31;14(17):4256. doi: 10.3390/cancers14174256 (PMC9454738; doi:10.3390/cancers14174256)
Supplement: Supplementary file 1 [file cancers-14-04256-s001.zip › Supplementary Methods.pdf]

SUPPLEMENTARY METHODS

Synthesis of Ketoconazole derivatives

KCZ-3 (Compound 3)

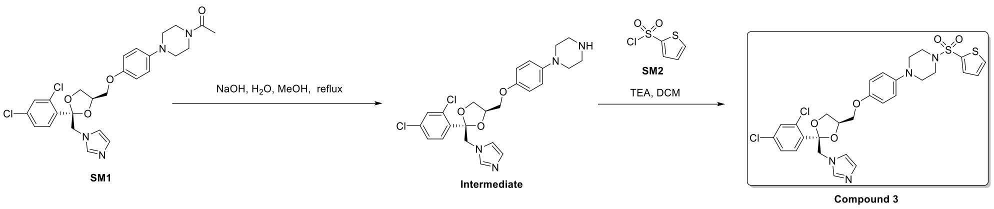

Synthesis of 1-(4-(((2S,4R)-2-((1H-imidazol-1-yl)methyl)-2-(2,4-dichlorophenyl)-1,3-dioxolan-4-yl)methoxy)phenyl)piperazine (Intermediate)

A solution of **SM1** (23.5 g, 40 mmol) and NaOH (8 g, 200 mmol) in MeOH/H_2O (100 mL/100 mL) was stirred at 70℃ for 3 h. The reaction mixture was filtered, and concentrated under vacuum, and extracted with Ethyl Acetate (100 mL x 3), the combined organic layer was washed with brine (50 mL x 3), then dried over with anhydrous Na_2SO_4. After filtration, the solution was concentrated under vacuum to give the crude product, which was purified by CombiFlash to give **Intermediate** (21.2 g, 98%) as a white solid. LC-MS $(M+H)^+$ = 489.

Synthesis of 1-(4-(((2S,4R)-2-((1H-imidazol-1-yl)methyl)-2-(2,4-dichlorophenyl)-1,3-dioxolan-4-yl)methoxy)phenyl)-4-(thiophen-2-ylsulfonyl)piperazine (Compound 3)

To a solution of **Intermediate** (100 mg, 0.205 mmol), triethylamine (31 mg, 0.308 mmol) in DCM (5 mL) was added dropwise to a solution of thiophene-2-sulfonyl chloride (56 mg, 0.308 mmol) in DCM (5 mL). The reaction mixture was stirred at room temperature for 3 hours. Then the reaction mixture was diluted with H_2O (10 mL), and extracted with Ethyl Acetate (10 mL x 3), the combined organic layer was washed with brine (10 mL x 3), then dried over with anhydrous Na_2SO_4. After filtration, the solution was concentrated under reduced pressure to give the crude product, which was purified by prep-HPLC to give ***Compound 3*** (45 mg, 35%). LC-MS $(M+H)^+$ = 635.

^{1}H NMR (400 MHz, $CDCl_3$) δ 8.76 (s, 1H), 7.73 (d, *J* = 8.4 Hz, 1H), 7.63 - 7.59 (m, 2H), 7.54 (d, *J* = 1.2 Hz, 1H), 7.40 - 7.30 (m, 4H), 7.24 - 7.21 (m, 2H), 6.85 (d, *J* = 7.2 Hz, 2H), 4.69 (q, *J* = 14.7 Hz, 2H), 4.42 (m, 1H), 3.96 (m, 1H), 3.90 - 3.75 (m, 2H), 3.71 (m, 1H), 3.52 (br, 8H).

KCZ-4 (Compound 4)

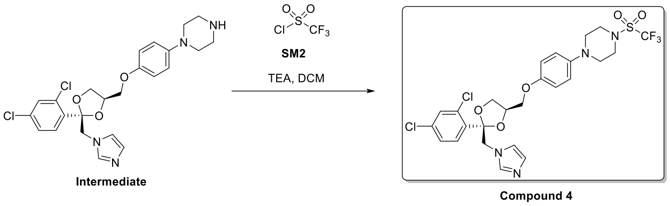

Synthesis of 1-(4-(((2S,4R)-2-((1H-imidazol-1-yl)methyl)-2-(2,4-dichlorophenyl)-1,3-dioxolan-4-yl)methoxy)phenyl)-4-((trifluoromethyl)sulfonyl)piperazine (Compound 4)

To a solution of **Intermediate** (100 mg, 0.205 mmol), triethylamine (31 mg, 0.308 mmol) in DCM (5 mL) was added dropwise a solution of trifluoromethanesulfonyl chloride (52 mg, 0.308 mmol) in DCM (5 mL). The reaction mixture was stirred at room temperature for 3 h. Then the reaction mixture was diluted with H_2O (10 mL), and extracted with Ethyl Acetate (10 mL x 3), the

combined organic layer was washed with brine (10 mL x 3), then dried over with anhydrous Na_2SO_4. After filtration, the solution was concentrated under reduced pressure to give the crude product, which was purified by prep-HPLC to give ***Compound 4*** (43 mg, 35%). LC-MS $(M+H)^+$ = 621.
^{1}H NMR (400 MHz, $CDCl_3$) δ 8.71 (s, 1H), 7.65 (d, *J* = 8.4 Hz, 1H), 7.52 (s, 1H), 7.40 - 7.30 (m, 3H), 7.24 - 7.20 (m, 1H), 7.11 - 7.05 (m, 1H), 6.75 (d, *J* = 8.0 Hz, 1H), 4.65 (q, *J* = 14.7 Hz, 2H), 4.39 (m, 1H), 3.93 – 3.75 (m, 3H), 3.70 - 3.50 (m, 5H), 3.24 (br, 4H).

KCZ-5 (Compound 5)

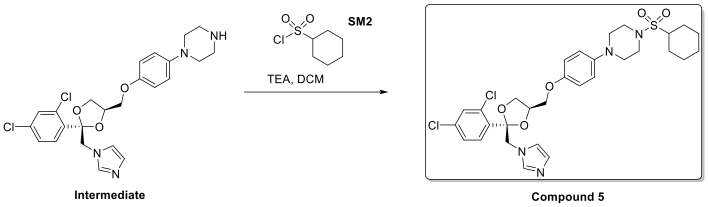

Synthesis of 1-(4-(((2S,4R)-2-((1H-imidazol-1-yl)methyl)-2-(2,4-dichlorophenyl)-1,3-dioxolan-4-yl)methoxy)phenyl)-4-(cyclohexylsulfonyl)piperazine (Compound 5)
To a solution of **Intermediate** (100 mg, 0.205 mmol), triethylamine (31 mg, 0.308 mmol) in DCM (5 mL) was added dropwise a solution of cyclohexanesulfonyl chloride (56 mg, 0.308 mmol) in DCM (5 mL). The reaction mixture was stirred at room temperature for 3 h. Then the reaction mixture was diluted with H_2O (10 mL), and extracted with Ethyl Acetate (10 mL x 3), the combined organic layer was washed with brine (10 mL x 3), then dried over with anhydrous Na_2SO_4. After filtration, the solution was concentrated under reduced pressure to give the crude product, which was purified by prep-HPLC to give ***Compound 5*** (60 mg, 46%). LC-MS $(M+H)^+$ = 635.
^{1}H NMR (400 MHz, $CDCl_3$) δ 8.69 (s, 1H), 7.64 (d, *J* = 8.4 Hz, 1H), 7.53 (d, *J* = 2.1 Hz, 1H), 7.36 (dd, *J* = 8.4, 2.1 Hz, 1H), 7.32 (d, *J* = 1.2 Hz, 1H), 7.24 - 7.17 (m, 3H), 6.83 (t, *J* = 6.3 Hz, 2H), 4.67 (q, *J* = 14.7 Hz, 2H), 4.47 - 4.35 (m, 1H), 3.95 (m, 1H), 3.84 (m, 1H), 3.77 - 3.68 (m, 6H), 3.40 - 3.30 (m, 4H), 2.98 (m, 1H), 2.16 (m, 2H), 1.92 (m, 2H), 1.74 (m, 1H), 1.54 (m, 2H), 1.27 (m, 3H).

KCZ-6 (Compound 6)

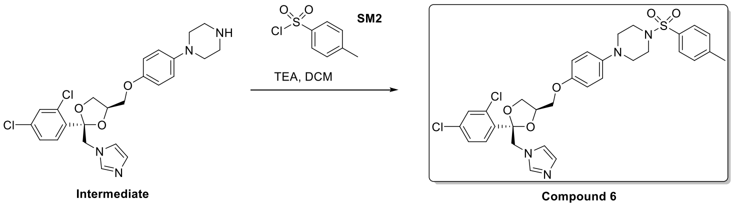

Synthesis of 1-(4-(((2S,4R)-2-((1H-imidazol-1-yl)methyl)-2-(2,4-dichlorophenyl)-1,3-dioxolan-4-yl)methoxy)phenyl)-4-tosylpiperazine (Compound 6)
To a solution of **Intermediate** (100 mg, 0.205 mmol), triethylamine (31 mg, 0.308 mmol) in DCM (5 mL) was added dropwise a solution of 4-methylbenzenesulfonyl chloride (59 mg, 0.308 mmol) in DCM (5 mL). The reaction mixture was stirred at room temperature for 3 h. Then the reaction mixture was diluted with H_2O (10 mL), and extracted with Ethyl Acetate (10 mL x 3), the combined organic layer was washed with brine (10 mL x 3), then dried over with anhydrous Na_2SO_4. After filtration, the solution was concentrated under reduced pressure to give the crude

product, which was purified by prep-HPLC to give ***Compound 6*** (KCZ-6; 65 mg, 49%). LC-MS $(M+H)^+$ = 643.
^{1}H NMR (400 MHz, MeOD) δ 8.98 (s, 1H), 7.71-7.62 (m, 2H), 7.53 (d, *J* = 2.1 Hz, 2H), 7.48 – 7.40 (m, 5H), 6.97 (d, *J* = 8.4 Hz, 2H), 6.79 (d, *J* = 8.8 Hz, 2H), 4.86 – 4.82 (m, 1H), 4.41 (s, 1H), 3.98 – 3.75 (m, 4H), 3.64 – 3.55 (m, 1H), 3.16 (br, 8H), 2.48 (s, 3H).

KCZ-7 (Compound 7)

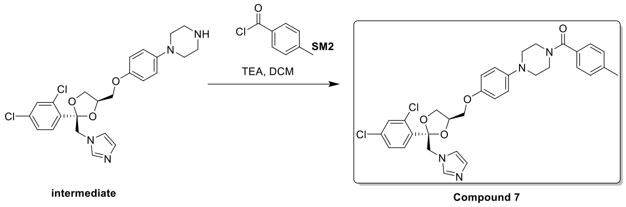

Synthesis of (4-(4-(((2S,4R)-2-((1H-imidazol-1-yl)methyl)-2-(2,4-dichlorophenyl)-1,3-dioxolan-4-yl)methoxy)phenyl)piperazin-1-yl)(p-tolyl)methanone (Compound 7)
To a solution of **Intermediate** (100 mg, 0.205 mmol), triethylamine (31 mg, 0.308 mmol) in DCM (5 mL) was added dropwise a solution of 4-methylbenzoyl chloride (48 mg, 0.308 mmol) in DCM (5 mL). The reaction mixture was stirred at room temperature for 3 h. Then the reaction mixture was diluted with H_2O (10 mL), and extracted with Ethyl Acetate (10 mL x 3), the combined organic layer was washed with brine (10 mL x 3), then dried over with anhydrous Na_2SO_4. After filtration, the solution was concentrated under reduced pressure to give the crude product, which was purified by prep-HPLC to give ***Compound 7*** (85 mg, 69%). LC-MS $(M+H)^+$ = 607.
^{1}H NMR (400 MHz, MeOD) δ 8.98 (t, *J* = 1.3 Hz, 1H), 7.76 (d, *J* = 8.5 Hz, 1H), 7.61 (t, *J* = 1.7 Hz, 2H), 7.50 - 7.41 (m, 2H), 7.39 - 7.34 (m, 2H), 7.30 (d, *J* = 7.9 Hz, 2H), 7.13 - 7.07 (m, 2H), 6.87 - 6.81 (m, 2H), 4.91- 4.83 (m, 2H), 4.41 (dq, *J* = 7.3, 5.4 Hz, 1H), 3.85 - 3.64 (m, 8H), 3.21 (s, 4H), 2.40 (s, 3H).

KCZ-10 (Compound 10)

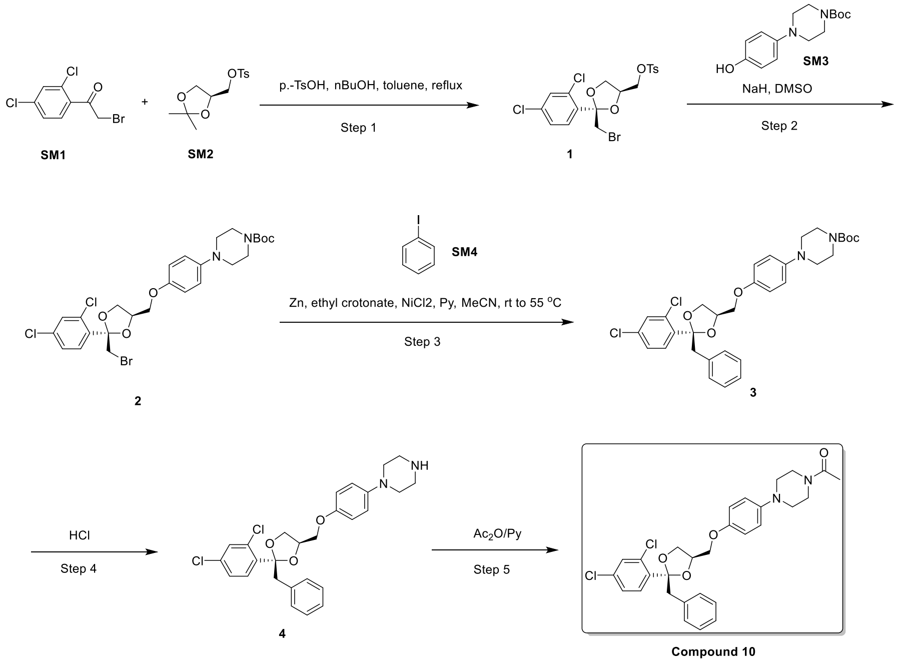

Synthesis of ((2S,4S)-2-(bromomethyl)-2-(2,4-dichlorophenyl)-1,3-dioxolan-4-yl)methyl 4-methylbenzenesulfonate (1)

A mixture of **SM1** (1.5 g, 5.6 mmol) and *p*-TsOH-H_2O (1.9 g, 9.96 mmol) in toluene (30 mL) was dried by reflux through a Dean-Stark trap for 1.5 h. A solution of **SM2** (2.0 g, 7.0 mmol) in toluene (20 mL) was similarly dried. Both reaction solutions were cooled to room temperature, and n-BuOH (10 mL) was added to the **SM2** solution. The resulting solutions were combined and heated at reflux through a fresh Dean-Stark trap containing 4-A molecular sieve. After 5 h, the mixture was cooled to room temperature and Et_3N (2.8 mL, 20 mmol) added. The mixture was stirred for 15 mins and then evaporated to dryness. The residue was partitioned between saturated aqueous $NaHCO_3$ and Ethyl Acetate. The organic layer was dried and evaporated, and the residue was purified by CombiFlash to give product **1** (1.1 g, 39%).

Synthesis of tert-butyl 4-(4-(((2S,4R)-2-(bromomethyl)-2-(2,4-dichlorophenyl)-1,3-dioxolan-4-yl)methoxy)phenyl)piperazine-1-carboxylate (2)

To a suspension of NaH (105 mg, 60% dispersion in oil, 2.62 mmol) in dry DMSO (10 mL) was added **SM3** (437 mg, 1.57 mmol). The mixture was stirred at room temperature for 1 h after which **1** (650 mg, 1.31 mmol) was added. The reaction mixture was heated at 80 ^oC for 5 h, cooled to room temperature, and poured into water. The mixture was extracted with Ethyl Acetate (20 mL x 3), and the combined organic phase was dried and evaporated to dryness. Purification by CombiFlash gave product **2** (550 mg, 70%). LC-MS $(M+H)^+$ = 601.

Synthesis of tert-butyl 4-(4-(((2R,4R)-2-benzyl-2-(2,4-dichlorophenyl)-1,3-dioxolan-4-yl)methoxy)phenyl)piperazine-1-carboxylate (3)

To a stirred slurry of Zn (162 mg, 2.5 mmol) in pyridine (0.5 mL) was added ethyl crotonate (86 mg, 0.75 mmol) at room temperature. Under stirring vigorously, $NiCl_2$ (32 mg, 0.25 mmol) was added to the above mixture. Then the temperature was increased to 55°C and stirring was continued for 15 mins. The resulting red–brown complex was cooled to room temperature, to which the solution of **SM4** (170 mg, 0.83 mmol) and **2** (500 mg, 0.83 mmol) in CH_3CN (8 mL) was added dropwise over a 10-min period. After 8 h, the mixture was filtered with a short plug (elution with 30 mL Et_2O) and washed with HCl (1 N), water and brine, dried over with anhydrous Na_2SO_4, filtered and concentrated. The crude product was purified by CombiFlash to give product **3** (250 mg, 50%). LC-MS $(M+H)^+$ = 599.

Synthesis of 1-(4-(((2R,4R)-2-benzyl-2-(2,4-dichlorophenyl)-1,3-dioxolan-4-yl)methoxy)phenyl)piperazine (4)

A solution of **3** (240 mg, 0.40 mmol) in 1M HCl/Dioxane (5 mL) was stirred at 0 ^oC for 1 h. The solution was concentrated under reduced pressure to give product **4** (150 mg, 75%). LC-MS $(M+H)^+$ = 499.

Synthesis of 1-(4-(4-(((2R,4R)-2-benzyl-2-(2,4-dichlorophenyl)-1,3-dioxolan-4-yl)methoxy)phenyl)piperazin-1-yl)ethan-1-one (Compound 10)

To a mixture of Ac_2O (2 mL) and pyridine (2 mL) on ice bath was added **4** (130 mg, 0.26 mmol). The reaction mixture was stirred at 0 °C for 1 h. Then the reaction mixture was concentrated under reduced pressure to give the crude product, which was purified by prep-HPLC to give ***Compound 10*** (75 mg, 53%). LC-MS $(M+H)^+$ = 541.

^{1}H NMR (400 MHz, $CDCl_3$) δ 7.49-7.40 (m, 2H), 7.34-7.30 (m, 2H), 7.26-7.16 (m, 6H), 6.80 (d, *J* = 8.0 Hz, 2H), 4.30 – 4.27 (m, 1H), 4.10 (s, 2H), 3.90 (s, 2H), 3.77 (m, 2H), 3.60 – 3.55 (m, 1H), 3.58 – 3.34 (m, 6H), 3.24 – 3.20 (m, 1H), 2.21 (s, 3H).

Cell-based chemical screens

Isogenic MDA-MB-231 and MDA-MB-231BRM cell lines stably expressing the control vector, GLI1-, or tGLI1-expression vectors, established in our previous study (1); MCF10A, HepG2, or immortalized human astrocytes were cultured in their respective culture media. Cells were harvested during the exponential growth phase, seeded at 2-4x10^3 cells per well in 96-well white bottom plates (Greiner Bio-One 655083), and incubated at 37°C, 5% CO_2 for 24 h. Cells were subsequently treated with test compound or vehicle control for 48 h. The final concentration of DMSO (vehicle) was 1% for all treatments. Viability was determined using the CellTiter-Blue® Cell Viability Assay (Promega G8080) according to the manufacturer's instructions.

Colony formation assay
Isogenic MDA-MB-231 and MDA-MB-231BRM cell lines in the exponential growth phase were harvested and seeded at 250 cells per well into 12-well plates (Corning 3513) and allowed to grow for 24 h. Culture medium was subsequently replaced with fresh media containing KCZ or vehicle control (1% DMSO). Treatment medium was replaced every 48 h during the 10-day treatment window. Cells were washed once with phosphate-buffered saline (PBS) before being stained for 1 h with 1.5% Crystal Violet (Millipore Sigma HT90132) diluted in 20% ethanol. Plates were washed with tap water and allowed to dry before colonies were counted.

Mammosphere assay
Adherent cells were harvested and seeded at a density of 1-4x10^3 cells per well in 24-well ultra-low attachment plates (Corning 3473) with Dulbecco's modified Eagle's medium/F12 (Gibco 11320033) containing 2% B27 (Gibco 17504044), 20 ng/mL recombinant EGF (Millipore Sigma PHG0311), 4 µg/mL insulin (Millipore Sigma 12585014), and 100 ng/mL recombinant Sonic Hedgehog protein (SHH, Millipore Sigma GF174). Beginning 24 h after seeding, mammospheres were treated with vehicle (1% DMSO), KCZ, or KCZ derivatives. Mammospheres were cultured for 7–14 days and supplemented with 100 µL of fresh treatment prepared in mammosphere medium every 48 h. The number of spheres with diameter of at least 100 µm were counted under 5× objective.

Selective knockdown of tGLI1 using antisense oligonucleotides (AS-ON)
Control or tGLI1 specific locked nucleic acid (LNA) AS-ONs were custom designed and purchased from Qiagen. Sequence for the negative control LNA AS-ON is /56-FAM/*A*A*C*A*C*G*T*C*T*A*T*A*C*G*C. BLAST analysis did not show binding of the control to any gene. Sequence for the tGLI1-targeting AS-ON is /56-FAM/$^{+}$C^{+}A^{+}A^{+}CT*T*G*A*C*T*T*C^{*+}T^{+}G^{+}TC. Phosphorothioated bases are indicated by * whereas LNA bases are labeled by +. Knockdown of tGLI1 was conducted as described previously (1,2). Briefly, BT-20 cells were transfected for 48 h with 100 nM control or tGLI1 AS-ON using Lipofectamine 2000 (Invitrogen 11668027). Cells were subsequently harvested for quantitative PCR or seeded for mammosphere assays.

Quantitative RT-PCR
RNeasy Mini Kit (Qiagen 74104) was used to isolate total RNA and cDNA was produced from 1 µg total RNA using the Superscript III First-Strand cDNA synthesis system (Invitrogen 18080044). Quantitative PCR was carried out as previously described (2,3) using primers described in Supplementary Table 1.

Animal studies
Female nude mice of 6-7 weeks of age (Charles River) were housed in a pathogen-free facility of the Animal Research Program at Wake Forest School of Medicine (WFSM) under a 12:12-h light/dark cycle and fed irradiated rodent chow *ad libitum*. Animal handling and procedures were approved by the WFSM Institutional Animal Care and Use Committee (IACUC). In the tumor

prevention model, mice received a single 100 µL intraperitoneal treatment of either vehicle or 50 mg/kg KCZ dissolved in 100% polyethylene glycol 300 (PEG-300, Sigma 202371) 24 h prior to intracardiac inoculation with $2x10^5$ exponentially growing SKBRM-tGLI1 cells in 100 µL ice-cold PBS. Successful inoculations were confirmed by visualization of brain bioluminescent signal within 60 minutes following inoculation; otherwise, mice were immediately sacrificed. Tumor progression was monitored with biweekly bioluminescent imaging (BLI) in which xenograft-bearing mice were intraperitoneally injected with 100 mg/kg d-luciferin (Perkin Elmer 122799) and imaged using the IVIS Lumina LT Series III imager (Perkin Elmer). Treatments were administered three times per week until study termination. Tumor burden was analyzed by quantifying BLI signal in each region-of-interest measured in total flux (p/s) with the Living Image software version 4.7.2 (Perkin Elmer). For the tumor treatment model, mice were intracardially inoculated with $2x10^5$ exponentially growing SKBRM-GLI1 or SKBRM-tGLI1 cells. Successfully inoculated mice were randomized into vehicle or drug treatment groups (50 mg/kg KCZ-5, KCZ-7, or KCZ dissolved in PEG-300; 50 mg/kg ITZ dissolved in 10% N,N-Dimethylacetamide (DMAc, Sigma ARK2190) and 90% PEG-300) with treatment beginning 13 days after inoculation. Tumor growth was monitored as described for the tumor prevention model.

Drug quantification in mouse serum and tissue samples

At the time of sacrifice, mice were overdosed with 200 µL of a 114 mg/kg ketamine, 17 mg/kg xylazine mixture. Mice were exsanguinated by intracardiac bleed prior to confirmation of euthanasia by cervical dislocation. Serum was collected by allowing the blood to clot at room temperature for 30 minutes. Serum was separated via centrifugation at 2000 x *g* for 15 minutes at 4°C and the supernatant collected. Brain samples were rapidly excised and washed three times in ice-cold PBS before being flash frozen in the liquid nitrogen vapor phase. Both sample types were stored at -80°C until mass spectrometry. At the time of analysis, samples were thawed and 50 µL of serum was extracted with 200 µL of cold methanol (Optima, Thermo Scientific, Waltham, MA, USA), vortexed, and allowed to incubate on ice for at least 1 h. Samples were then centrifuged at 16 000 x *g* for 10 minutes and diluted appropriately in methanol. A portion of the thawed tissue was removed and weighed into a bead mill homogenization tube containing 1.4 mm ceramic beads (Thermo Scientific, Waltham, MA, USA). Tissue was homogenized in a Bead Ruptor 24 (OMNI International, Kennesaw, Georgia) bead mill homogenizer after the addition of 1 mL of 80% methanol in water (Optima, Thermo Scientific, Waltham, MA, USA). Samples were then allowed to incubate on ice for at least 1 h, centrifuged at 16 000 x *g* for 10 minutes, and diluted appropriately in methanol.

Sample extracts were quantified with a Shimadzu Nexera UHPLC system coupled with a Shimadzu LCMS-8050 operated with a dual ion source (DUIS) (Kyoto, Japan). Separation was conducted with a Zorbax Eclipse Plus C18 RRHD column (1.8 µm, 2.1 x 100 mm; Agilent Technologies, Santa Clara, CA USA) under gradient conditions at a 0.4 mL/min flow rate. Mobile phases A and B were 0.1% formic acid (Thermo Scientific, Waltham, MA, USA) in water and acetonitrile (Optima, Thermo Scientific, Waltham, MA, USA), respectively. The initial gradient began at 10% B for the first minute before increasing to 95% B between 1 and 4 minutes. The gradient was held at 95% B for 1 minute, reduced to 10% B between 5 and 5.1 minutes, and held at that point until ending the method at 6 minutes. Analytes underwent ionization in the DUIS source with the following conditions: nebulizing gas flow of 2 L/min, heating gas flow of 10 L/min, interface temperature of 300°C, DL temperature of 250°C, heat block temperature of 400°C, and a drying gas flow of 10 L/min. KCZ, KCZ-5, and KCZ-7 were analyzed with three m/z transitions each in positive ESI mode: KCZ 530.90 > 489.15, 530.90 > 82.10, 530.90 > 177.10; KCZ-5 635.20 > 489.20, 635.20 > 459.20, 635.20 > 55.05; KCZ-7 606.90 > 119.05, 606.90 > 91.10, 606.90 > 589.25.

Alanine transaminase assay for liver toxicity

The Alanine Transaminase Colorimetric Assay kit (Cayman Chemical 700260) was used to determine alanine transaminase activity according to the manufacturer's instructions. Briefly, serum samples were thawed on ice and centrifuged at 2 000 x *g* for 15 minutes at 4°C before transferring to a sterile 1.5 mL microcentrifuge tube. Cleared serum samples and kit components were equilibrated to room temperature before assembling the assay plate with 20 µL positive control or sample. Absorbance at 340 nm was measured every minute for 10 minutes at 37°C. The change in absorbance (ΔA_{340}) per minute was determined for each sample. ALT activity (U/L) was then determined with the equation:

$$ALT\ activity\ (U/L) = \left[\frac{\Delta A_{340}/min \times 0.21\ mL}{4.11\ mM^{-1} \times 0.02\ mL}\right] \times\ 1\ 000$$

Samples were run in technical duplicate and five samples per treatment group were assayed using a SpectraMax iD3 plate reader (Molecular Devices).

Immunohistochemistry (IHC)

Brain specimens from the intracardiac animal studies were flash frozen in Optimal Cutting Temperature compound following resection. Brain slices were sectioned at 10 µm thickness and the RFP signal was used to confirm presence of brain metastases. IHC was performed as previously described (1). IHC antibodies included Ki-67 (Cell Signaling, 9027, 1:400), VEGF-A (A-20) (Santa Cruz, sc-152, 1:25), mCD31 (BD Bioscience, 550274, 1:100), and a rabbit polyclonal tGLI1-specific antibody (YenZym Antibodies, 1:50) designed and validated in our laboratory (1-6). Stained sections were imaged using the colorimetric protocol and 20X objective of an ImageXpress Pico Automated Cell Imaging System (Molecular Devices). Histologic scores (H-scores) were calculated using the equation:

$$H\ score = A\ \times B$$

where A represents percent positivity (A%, A = 1-100) and B represents intensity (B = 0-3). Microvessel density (MVD) was measured by counting the number of vessels per area and computed by the equation:

$$MVD = vessels/mm^2$$

Production of tGLI1 recombinant protein

The full-length tGLI1 coding sequence was cloned into a modified pET28 expression vector (pLM303-tGLI1) containing an N-terminal maltose-binding protein (MBP) tag and an intervening rhinovirus 3C protease cleavage site. The expression construct was transformed into BL21(DE3) competent *E. coli* (Sigma CMC0014) and grown in Luria-Bertani (LB) medium at 37°C with shaking to an OD_{600} = 0.6. Cultures were induced with 0.3 mM isopropyl-β-D-thiogalactoside (IPTG) and allowed to express protein at 16°C overnight. Harvested bacteria were resuspended in bacterial resuspension buffer (50 mM Tris pH 7.5, 300 mM NaCl, 1 mM $MgCl_2$, 0.1 mM EDTA, 10% glycerol) and cOmplete protease inhibitor cocktail (Roche), and lysed using an Avestin Emulsiflex-C5 cell homogenizer. Cell debris was cleared by centrifugation at 10,000 rpm at 4°C for 30 min, and the cleared lysate was passed over amylose high flow resin (New England Biolabs) and washed with at least 3 column volumes of amylose column buffer (ACB) (50 mM Tris pH 7.5, 250 mM NaCl, 2 mM $MgCl_2$, 0.1 mM EDTA, 2 mM DTT, 10% glycerol). The bound MBP-tGLI1 protein was eluted with ACB plus 20 mM maltose.

The desired fractions were pooled and dialyzed overnight against heparin column buffer (HCB) (50 mM Tris pH 7.5, 100 mM NaCl, 2 mM $MgCl_2$, 0.1 mM EDTA, 2 mM DTT, 5% glycerol) containing HRV 3C PreScission Protease (GE Biosciences) to cleave the MBP tag. The tGLI1 recombinant protein was separated from the cleaved MBP using a Heparin HiTrap (GE Healthcare Life Sciences) and eluted using a linear gradient of HCB plus 2 M NaCl. Fractions containing tGLI1 were pooled, spin concentrated using a Vivaspin 20 (Vivaproducts VS2002), and aliquots were frozen on dry ice, and stored at -80°C until use.

Electrophoretic mobility shift assay
Approximately 600 ng of recombinant STAT3 (Creative Biomart, STAT3-29823TH), GLI1 (Creative Biomart, GLI1-312H), or N-tGLI1 protein was mixed with 5X binding buffer (50 mM Tris pH 7.5, 50 mM NaCl, 200 mM KCl, 5 mM $MgCl_2$, 10 mM EDTA, 5 mM DTT, 250 µg/mL BSA, 25% glycerol), 50 ng/µL poly dI·dC (Sigma P4929), and 5 pmol 6FAM-labeled dsDNA oligo (Integrated DNA Technologies) in a total reaction volume of 20 µL. The oligos were ordered as dsDNA from IDT with the sequences /56-FAM/CGAAGAGACCACCCAGGTAGCT and /56-FAM/AGCTACCTGGGTGGTCTCTTCG; the GLI1 consensus binding sequence is underlined. Binding reactions were incubated on ice for 20 min before electrophoresis on 6% (19:1) acrylamide:bisacrylamide TBE gels using 0.5X TBE + 2.5% glycerol running buffer at 80V. For drug disruption studies, the binding buffer, protein, and treatment were combined and incubated for 30 min on ice or room temperature for GLI1 or N-tGLI1, respectively. Final treatment concentrations were 1% DMSO, 100 µM KCZ, or 100 µM KCZ-7. After addition of the dsDNA oligo and poly dI·dC, reactions were incubated for an additional 30 minutes before electrophoresis. Gels were imaged using the fluorescein module on a ChemiDoc MP (BioRad).

Chromatin immunoprecipitation
SKBRM cells in the exponential growth phase were transfected with GLI1 or tGLI1 expression plasmid and a GLI1-binding site driven luciferase construct 8x3'GLI1 generously provided by Dr. Hiroshi Sasaki (Osaka University) (7). After 24 h, cells were treated with 1% DMSO, KCZ, or KCZ-7 for 20 h. Cells were stimulated with 100 ng/mL SHH for 4 h prior to crosslinking with 1% formaldehyde. Excess formaldehyde was quenched with 0.125 M glycine and the ChIP assay was carried out as described previously using the ChIP assay kit from EMD Millipore (Cat No. 17-371) (2). GLI1 and tGLI1 cell lysates were immunoprecipitated using a GLI1 antibody (CST 2643) that recognizes both GLI1 and tGLI1 and has been successfully used for ChIP (8). Mouse normal IgG served as negative immunoprecipitation controls and input chromatin was used as loading controls for quantitative RT-PCR. The primers used for detection of the GLI1-binding site are 5'- GAGTCAGTGAGCGAGGAAG-3' and 5'-GCCGGGCCTTTCTTTATGT-3'.

Western blotting
Immunoblotting was performed as previously described (1,2,6). Antibodies include GLI1 (Cell Signaling Technology/CST; 2643, 1:1000), custom-made tGLI1-specific antibody (Yenzyme, 1:1000), OCT4 (CST 2750 and CST 4286, 1:1000), Nanog (CST 4903, 1:1000), androgen receptor (CST 5153, 1:1000), α-Tubulin (Sigma T6074, 1:5000), β-actin (CST 3700, 1:5000), Vinculin (CST 13901, 1:5000). Densitometry was performed using ImageJ v1.53c (NIH, USA).

Promoter reporter assay
SKBR3 cells in the exponential growth phase were transfected with Vector, GLI1, or tGLI1 expression plasmid, 8x3'GLI1-luciferase reporter, and Renilla luciferase (pRL-TK) to control for transfection efficiency for firefly luciferase using XtremeGene HP (Roche). After 24 h, cells were treated with vehicle (1% DMSO) or increasing doses of KCZ or KCZ-7 for 20 h. Cells were stimulated with 100 ng/mL SHH for 4 h before cell lysates were harvested and luciferase activity was measured using a Firefly and Renilla luciferase kit (Biotium 30081) on a SpectraMax iD3

plate reader (Molecular Devices). Total transfection and drug treatment times were 48 and 24 h, respectively. Relative promoter activity was determined by normalizing the luciferase activity to the renilla control.

Statistical analysis

Data were analyzed and graphed using Prism 9.1 (GraphPad). Descriptive statistics are presented as mean ± SEM. Repeated measures ANOVAs with *post hoc* Bonferroni's or Dunnett's multiple comparison test was performed using Prism 9.1 and used to analyze differential drug effects in the chemical screen, colony formation, mammosphere formation, and gene expression assays. Student's *t*-test, one-way ANOVA with Dunnett's or Tukey's multiple comparison test, and nonlinear regression analyses were also performed using Prism.

References

1. Sirkisoon SR, Carpenter RL, Rimkus T, Doheny D, Zhu D, Aguayo NR*, et al.* TGLI1 transcription factor mediates breast cancer brain metastasis via activating metastasis-initiating cancer stem cells and astrocytes in the tumor microenvironment. *Oncogene* **2020**;39:64-78.
2. Rimkus TK, Carpenter RL, Sirkisoon S, Zhu D, Pasche BC, Chan MD*, et al.* Truncated Glioma-Associated Oncogene Homolog 1 (tGLI1) Mediates Mesenchymal Glioblastoma via Transcriptional Activation of CD44. *Cancer research* **2018**;78:2589-600.
3. Zhu H, Carpenter RL, Han W, Lo HW. The GLI1 splice variant TGLI1 promotes glioblastoma angiogenesis and growth. *Cancer letters* **2014**;343:51-61.
4. Carpenter RL, Paw I, Zhu H, Sirkisoon S, Xing F, Watabe K*, et al.* The gain-of-function GLI1 transcription factor TGLI1 enhances expression of VEGF-C and TEM7 to promote glioblastoma angiogenesis. *Oncotarget* **2015**;6:22653-65.
5. Han W, Carpenter RL, Lo H-W. TGLI1 Upregulates Expression of VEGFR2 and VEGF-A, Leading to a Robust VEGF-VEGFR2 Autocrine Loop and Cancer Cell Growth. *Cancer Hallmarks* **2013**;1:28-37.
6. Sirkisoon SR, Carpenter RL, Rimkus T, Anderson A, Harrison A, Lange AM*, et al.* Interaction between STAT3 and GLI1/tGLI1 oncogenic transcription factors promotes the aggressiveness of triple-negative breast cancers and HER2-enriched breast cancer. *Oncogene* **2018**;37:2502-14.
7. Sasaki H, Hui C, Nakafuku M, Kondoh H. A binding site for Gli proteins is essential for HNF-3beta floor plate enhancer activity in transgenics and can respond to Shh in vitro. *Development (Cambridge, England)* **1997**;124:1313-22.
8. Pietrobono S, Anichini G, Sala C, Manetti F, Almada LL, Pepe S*, et al.* ST3GAL1 is a target of the SOX2-GLI1 transcriptional complex and promotes melanoma metastasis through AXL. *Nature communications* **2020**;11:5865.
